# Ultrasonographic‐based predictive factors influencing successful return to racing after superficial digital flexor tendon injuries in flat racehorses: A retrospective cohort study in 469 Thoroughbred racehorses in Hong Kong

**DOI:** 10.1111/evj.12810

**Published:** 2018-02-23

**Authors:** R. Alzola, C. Easter, C. M. Riggs, D. S. Gardner, S. L. Freeman

**Affiliations:** ^1^ Oakham Veterinary Hospital Oakham Rutland UK; ^2^ Oxtex Ltd Witney Oxfordshire UK; ^3^ Veterinary Clinical Services Hong Kong Jockey Club Hong Kong; ^4^ School of Veterinary Medicine and Science University of Nottingham Loughborough Leicestershire UK

**Keywords:** horse, tendinopathy, SDFT, ultrasonography, predictive factors

## Abstract

**Background:**

Superficial digital flexor tendon (SDFT) injury is an important health and welfare concern in racehorses. It is generally diagnosed with ultrasonography, but predictive ultrasonographic features have not been reported.

**Objectives:**

To determine ultrasonographic features of forelimb SDFT injury at initial presentation in Thoroughbred racehorses that could predict a successful return to racing (completing ≥5 races).

**Study design:**

Retrospective cohort study.

**Methods:**

Digitised ultrasonographic images of 469 horses with forelimb SDFT injuries from the Hong Kong Jockey Club (2003–2014) were evaluated, using a previously validated ultrasonographic scoring system. Six ultrasonographic parameters were evaluated (type and extent of the injury, location, echogenicity, cross‐sectional area and longitudinal fibre pattern of the maximal injury zone [MIZ]), as well as horse signalment, retirement date and number of races before and after injury. Data were analysed by generalised linear regression with significance at P<0.05.

**Results:**

Cases were divided into two groups: 1) For cases of SDFT tendonitis with core lesions, cross‐sectional area at the MIZ was the most significant factor determining a successful return to racing (P = 0.03). If the lesion was <50% of the total cross‐sectional area, horses had 29–35% probability of successfully racing again, but if it was ≥50% this decreased to 11–16%. 2) For cases of SDFT tendonitis without a core lesion, longitudinal fibre pattern at the MIZ best predicted a successful return to racing (P = 0.002); if the affected longitudinal fibre pattern was <75% of the total, horses had 49–99% probability of successfully return to racing, but if it was ≥75% this decreased to 14%.

**Main limitations:**

Prognostic information may not be applicable to other breeds/disciplines.

**Conclusions:**

This is the first study to describe ultrasonographic features of forelimb SDFT injuries at initial presentation that were predictive of successful return to racing. The outcomes will assist with early, evidence‐based decisions on prognosis in Thoroughbred racehorses.

## Introduction

Superficial digital flexor tendon (SDFT) tendonitis is common in racehorses 1, 2 and is likely to curtail their athletic career. Tendons have a limited ability to heal and their mechanical properties are degraded following injury, predisposing to re‐injury 3. The prevalence of SDFT tendonitis in flat Thoroughbred horses is between 3.4% and 11.1% 1, 4 and this injury is responsible for 14% of all retirements at the Hong Kong Jockey Club (HKJC) 2. Following any tendon injury, less than 50% of horses will return to racing, and 56% of those will re‐injure 5, 6.

Many different imaging modalities may be used to evaluate tendons. Ultrasonography is widely available and cost effective in equine practice, making it the main imaging technique for detection and assessment of tendonitis 7. SDFT injuries occur predominantly in the mid‐metacarpal region of the forelimbs 1 and may be concentrated in one or several locations in a single or multiple limbs. The central core region or the periphery of the tendon may be affected, although experimental studies have suggested a predisposition to the former 8, 9. The size of the lesion may vary in terms of the proportion of the cross‐sectional area of the tendon affected and the length of the injury. In addition, the degree of damage can vary from mild inflammation, associated with diffuse oedema of the tendon matrix, to complete fibre rupture affecting the echogenicity, cross‐sectional area and fibre alignment on ultrasonography. All these variables can potentially complicate the interpretation and recording of ultrasonographic findings in SDFT tendonitis. Ultrasonographic predictive values are widely used in multiple disciplines in human medicine but they are still underused in equine practice 10.

The objectives of the study were to: 1) describe the ultrasonographic features of SDFT tendonitis at initial presentation and 2) determine which features were the most valuable prognostic indicators in determining outcome. We hypothesised that initial ultrasonographic parameters of SDFT injury could predict the likelihood of horses successfully returning to racing (defined as completing ≥5 races).

## Materials and methods

### Cases

Digitised ultrasonographic records of 1161 horses with a forelimb SDFT injury sustained while training or racing at the HKJC between 2003 and 2014 were reviewed retrospectively. Inclusion criteria for the study were: horses with images of a complete ultrasonographic examination at the primary assessment (defined as a minimum of 10 images, including transverse views at seven specific levels and three longitudinal views at three levels through the metacarpus 11) and a full set of clinical data (including signalment, treatments, retirement date, and number of races before and after injury). Images were retrieved from a digital archive using an AGFA PACS system.^a^ Only data from the first ultrasonographic examination, performed within 1 week of an injury being initially documented, were selected for analysis.

### Evaluation of the SDFT injuries

**Figure 1:**
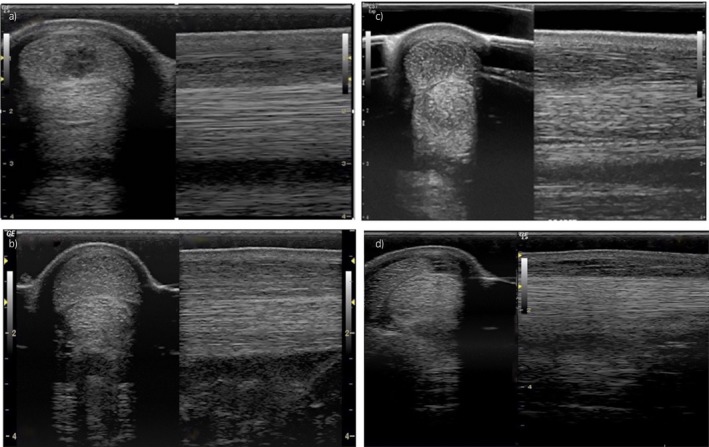
Transverse and longitudinal ultrasonographic images of SDFT injury: a) SDFT tendonitis with an anechoic core lesion, b) SDFT tendonitis with a hypoechoic core lesion, c) hypoechoic SDFT tendonitis without a core lesion and d) anechoic SDFT tendonitis without a core lesion.

The ultrasonographic images were reviewed and scored using a previously validated scoring system 12 by a single researcher (R.A.). Each ultrasonographic study was qualitatively assessed for the type of SDFT lesion (SDFT tendonitis with or without a core lesion, Fig 1), the length of the injury (number of zones affected; from 1 to ≥5 zones) and the location of the maximal injury zone (MIZ), defined as zones 1A–3C 13. Three ultrasonographic parameters within the MIZ were assessed semi‐quantitatively: a) lesion echogenicity (anechoic, hypoechoic or hyperechoic); b) proportion of SDFT cross‐sectional area affected (MIZ‐CSA %), categorised into one of four groups: 1) <25% of total tendon cross‐sectional area, 2) ≥25–50%, 3) ≥50–75% or 4) ≥75%; and c) proportion of disruption of the longitudinal fibre pattern (MIZ‐LFP %), categorised into one of five groups: 0) no disruption; 1) <25% disruption of the whole fibre pattern; 2) ≥25–50%; 3) ≥50–75% or 4) ≥75%. Other information, such as the affected limb (right, left or both) and, in cases of SDF tendonitis with core lesion, the location of the core (central, peripheral or both) and number of lesions (one or two) were also recorded.

### Outcome

To evaluate the outcome of each case, the following data were analysed: sex, age at time of injury, country of origin, racing data (number of races, distance and earnings before and after injury), received treatment, date of the first race following injury, and the reason and date for retirement. A successful return to racing was defined as horses that completed five or more races post‐injury 13.

### Data analysis

Sample size for the study was estimated after analysis of a preliminary cohort study of racehorses over a 5‐year period at the same location that were diagnosed with SDFT injury and subsequently retired or recovered and raced again. Of the 245 horses, 108 retired due to the initial injury with 93 successfully racing again (≥5 races). This gave the study an overall event rate of 44% for the primary outcome (raced successfully again or not). The null hypothesis was that ultrasonography of the initial injury site had no clinical diagnostic value for predicting the successful return of a racehorse to racing (i.e. a baseline event rate of 44%). We adopted a conservative approach and hypothesised that if ultrasonography at initial injury could better predict if a racehorse made a successful return to racing (≥5 races completed) by a 10‐unit change (e.g. from baseline event rate of 44% to 34%) between categories of SDFT injury (e.g. between categories of longitudinal fibre pattern affected <25% vs. ≥75%), this would be clinically significant. The sample size required with 90% probability (power) to detect a difference (two‐sided α of 0.05) was 203 cases in each arm of the study; a total of 406 cases were therefore required. Over the 10‐year period, clinical records of 469 cases in total were accessed, giving the study >85% power to observe a 10% difference in our primary outcome.

Descriptive data are presented as either mean (±s.d.) or median (+ interquartile range; IQR) for normal or skewed distributions respectively. Categorical data on ultrasonographic parameters of the limb (limb affected [right forelimb, left forelimb or both]), length of the injury [1 to ≥5 zones], location of the MIZ [1A–3C zone], location of the core lesion [central/peripheral/both], cross‐sectional area affected [grade], echogenicity and longitudinal fibre pattern affected [grade] of the MIZ) are presented as mean proportions (i.e. number [percent of total]) and analysed by χ^2^. Ultrasonographic parameters of the type of injury (SDFT tendonitis with or without core lesion) were analysed as two separate data sets with individual descriptors. Other factors related to horse (sex, weight and age at injury, country of origin and treatment) or to their racing career (number of races before and after injury and time to the first race post‐injury) were also included in the statistical models as potential confounders (i.e. as co‐variants, as appropriate). The statistical model was built by first testing potentially important factors through univariate analysis, including those contributing at P≤0.10 into a multivariate analysis (generalised linear regression) with a binomial error distribution and a logistic‐link function. With ‘successfully raced again’ as the response variable (binomial; yes/no), factors hypothesised to affect that response were initially included as explanatory factors (i.e. location of injury such as limb, number of zones affected, location of the MIZ, core or peripheral lesion, MIZ‐cross‐sectional area and MIZ‐longitudinal fibre alignment) and the final model fit assessed by backward stepwise analysis. Data are presented as predicted effect size ± s.e.d. Effects were deemed as significant when P<0.05. All data analysis was undertaken with GenStat v18.^b^

## Results

A total of 471 horses met the inclusion criteria but a complete data set was only available for 469 horses: 357 cases presented with SDFT tendonitis with a core lesion (Group 1) and 112 cases with SDFT tendonitis without a core lesion (Group 2). The average age of horses that presented with an SDFT injury was 4 (IQR = 3–5) years old (Supplementary Item 1). With the exception of one female horse in Group 1, all the horses in this study were male and predominantly geldings (Group 1: 95%; Group 2: 93%).

### Ultrasonographic features of the SDFT injury

#### Location of the lesion

**Figure 2:**
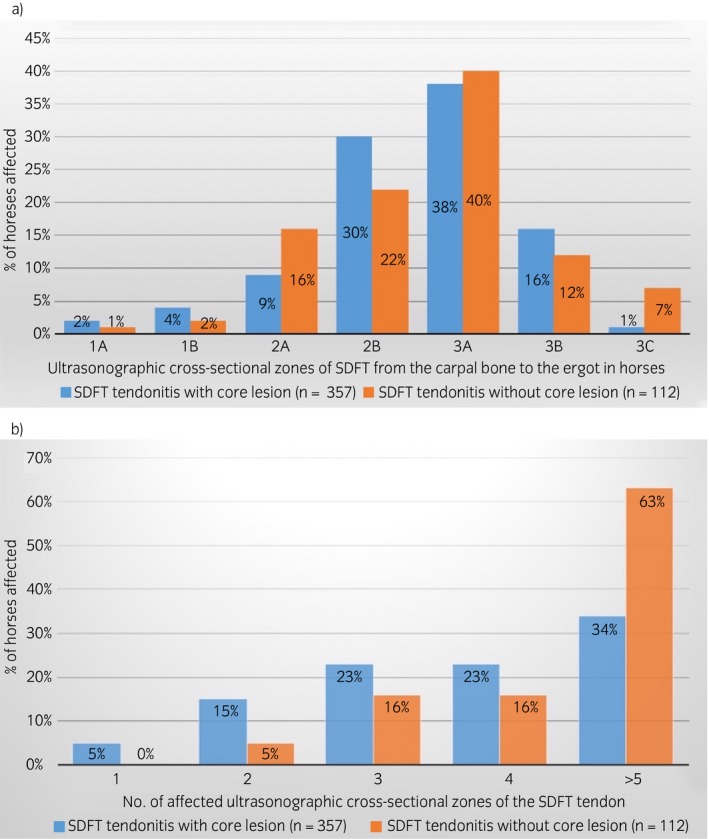
Ultrasonographic features of 469 flat racehorses with SDFT injury in Hong Kong (2003–2014): a) location of the maximal injury zone (MIZ) and b) number of affected cross‐sectional zones (length of the lesion).

SDFT lesions were significantly more common in the right vs. left forelimb in both groups (P<0.001) (Supplementary Item 2). Only a small number of cases had both limbs affected (Group 1: 20 [5.7%]; Group 2: 18 [16%]). In both groups, the mid‐metacarpal region (zones 2B and 3A) was the most common site of the maximal injury zone (MIZ; P<0.001) (Fig 2a).

#### Severity of the lesion

The length of tendon affected by the lesion varied significantly between the groups (P<0.001) (Fig 2b): 37.8% (n = 135/357) of horses in Group 1 had 2–3 zones affected compared with 21.4% (n = 24/112) in Group 2. The prevalence of horses that presented with an injury extending over more than three zones was, Group 1: 57.1% (n = 204/357), Group 2: 79.4% (n = 89/112). All injuries had either a moderate (hypoechoic) or severe (anechoic) reduction in echogenicity at the MIZ. Lesions graded as hypoechoic accounted for 6.4% in Group 1 and 69.6% in Group 2, while those graded as anechoic were 93.5% in Group 1 and 30.3% in Group 2.

#### Cross‐sectional area (MIZ‐CSA) and longitudinal fibre pattern (MIZ‐LFP) affected at the MIZ (Table 1)

Group 1: Small core lesions were significantly more common (P<0.001), affecting <25% of the MIZ‐CSA. For the fibre alignment (MIZ‐LFP), lesions affecting more than 75% of the MIZ‐LFP were significantly more common (P<0.001).

**Table 1:** Ultrasonographic scores of the cross‐sectional area (MIZ‐CSA) and longitudinal fibre pattern (MIZ‐LFP) affected at the maximal injury zone (MIZ) in 469 horses with SDFT injury in Hong Kong (2003–2014)

	SDFT tendonitis with core lesion (n = 357)		SDFT tendonitis without core lesion (n = 112)	
	No° of horses	%	No of horses	%
Longitudinal Fibre Pattern‐MIZ^a^				
Score 0 = 0%	0	0.0	0	0.0
Score 1 = <25%	39	10.9	1	0.0
Score 2 = ≥25–50%	98	27.4	29	25.8
Score 3 = ≥50–75%	92	25.7	30	26.7
Score 4 = ≥75%	128	35.8	52	46.4
Cross Sectional Area‐MIZ^a^				
Score 1 = <25%	198	55.4	15	13.3
Score 2 = ≥25–50%	111	31.0	37	33.0
Score 3 = ≥50–75%	38	10.6	36	32.1
Score 4 = ≥75%	10	2.8	24	21.4

a MIZ, Maximal injury zone.

Group 2: Lesions affecting at least 50% of the CSA were significantly more common (P<0.001) than lesions affecting <50% of the CSA. For fibre alignment (MIZ‐LFP), lesions affecting over 75% of the LFP at the MIZ were significantly more common (P<0.001) than those affecting less than 75% of the LFP‐MIZ.

### Treatments

Treatment options were varied and included use of a controlled exercise programme, NSAIDs, steroids, blistering, shockwave, ice, intralesional therapy (Platelet rich plasma, Stem cell therapy), surgery (i.e. desmotomy of the accessory ligament of the SDFT) or various combinations of these. The study was powered and designed to test the primary outcome (successfully raced again after SDFT injury) rather than the efficacy of a particular treatment regimen. Hence, interpretation of secondary outcomes such as treatments for SDFT are not within the scope of the study, but are included for interest and with a cautionary note in supplementary information (Supplementary Item 3).

### Racing outcome after injury

**Figure 3:**
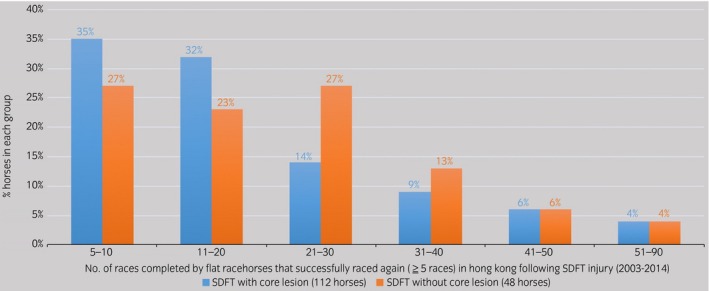
Number of races completed by flat racehorses that successfully raced again (≥5 races) in Hong Kong following a SDFT injury (2003–2014).

Group 1: Forty‐nine percent of horses (n = 175/357) raced again at least once, but only 31.3% (n = 112/357) raced five or more times and were considered to have had a successful return to racing; 51% of horses never raced again after the initial SDFT injury (n = 182/357) due to either the original injury itself or because of recurrence of injury at the same site. The average number of days from injury to retirement in this group was 94 days (IQR = 14–275). For horses that successfully raced again, the average number of days from injury to the first race was 196 (IQR = 103–285) days, similar to those horses that raced again less than five times, 194 (IQR = 115–288 days). Racehorses that successfully raced again participated in an average of 14 races (IQR = 9–24; Fig 3) over a period of 774 (IQR = 569–1084) days.

Group 2: While 53.5% (n = 60/112) of horses in this group raced again at least once, only 43.7% (n = 49/112) successfully raced again (i.e. ≥5 races). The average time to retirement of horses that did not race again was 174 (IQR = 15–323) days from injury. Horses that raced again successfully (≥5 races), undertook their first start 192 (IQR = 122–290) days post‐injury while those that raced ‘unsuccessfully’ (<5 starts) commenced racing again 165 (IQR = 51–292) days after injury. For horses in Group 2 making a successful return to racing, the average number of races they participated in was 20 (IQR = 9–29; Fig 3), and average time to retirement was 668 (IQR = 357–979) days.

### Factors influencing a successful return to racing

In Group 1, five factors at the time of the initial injury were found to significantly influence the chances of successfully returning to racing: age of the horse (P<0.001), number of starts prior to the injury (P<0.001), location of the core lesion (P = 0.02), MIZ‐CSA grade (P = 0.03) and MIZ‐echogenicity grade (P = 0.029). Despite investigating the same factors in Group 2, only two US parameters were significant; MIZ‐echogenicity (P<0.001) and MIZ‐LFP (P = 0.002). Further multivariate logistic regression analysis of factors important for a successful return to racing in Group 1 and Group 2 was performed separately. For Group 1, MIZ‐CSA (P = 0.03), MIZ‐echogenicity (P = 0.04) and core lesion location (P = 0.04), and for Group 2, MIZ‐LFP (P = 0.002) and MIZ‐echogenicity (P = 0.008) remained as significant influences. More specifically, a reduction in echogenicity (hypoechoic or anechoic) of the MIZ at the time of injury was found to be significant in both groups (Table 2). Predicting values for a successful return to racing (completing ≥5 races) are presented for MIZ‐CSA in Group 1 and MIZ‐LFP in Group 2 (Table 3).

**Table 2:** Multivariable logistic regression model of risk factors for a successful return to racing in 469 horses with SDFT injury in Hong Kong (2003–2014)

Variable	Univariate analysis	Wald	P‐value	Multivariate analysis	Wald	P‐value
	OR (95% CI)	Statistic		OR (95% CI)^b^	Statistic	
**Group 1: Racehorses with SDFT tendonitis with core lesion**						
Age at injury	0.73 (0.59–0.89)	8.92	0.003	–	–	–
Echogenicity (of MIZ)	Ref: Anechoic 1: 2.28 (0.93–5.55)	– 3.30	– 0.06	– 2.61 (1.10–6.78)	– 3.92	– 0.04
Cross‐sectional area (of MIZ)	Ref: <25% 1: 0.68 (0.40–1.15) 2: 0.29 (0.11–0.80) 3: 0.20 (0.02–1.65)	– 8.48	– 0.03	– 0.90 (0.46–1.74) 0.38 (0.12–1.18) 0.28 (0.03–2.57)	– 3.76	– 0.2
Longitudinal fibre pattern (of MIZ)	Ref: <25% 1: 0.98 (0.45–2.15) 2: 0.68 (0.30–1.54) 3: 0.44 (0.19–0.97)	– 8.16	– 0.04	– 0.93 (0.42–2.06) 0.74 (0.30–1.78) 0.65 (0.25–1.67)	– 1.09	– 0.7
**Group 2: Racehorses with SDFT tendonitis without core lesion**						
Age at injury	0.68 (0.48–0.96)	4.69	0.03	–	–	–
Echogenicity (of MIZ)	Ref: Anechoic 12.7 (3.53–45.9)	– 15.1	– <.001	– 16.8 (1.77–161)	– 6.00	– 0.01
Cross‐sectional area (of MIZ)	Ref: <25% 1: 2.49 (0.58–10.6) 2: 4.20 (1.00–17.6) 3: 3.97 (0.86–18.1)	– 4.53	– 0.20	– 0.13 (0.01–1.87) 0.61 (0.04–7.69) 0.47 (0.03–6.59)	– 5.40	– 0.2
Longitudinal fibre pattern (of MIZ)	Ref: 25–49%^a^ 3: 0.37 (0.12–1.12) 4: 0.14 (0.05–0.40)	– 13.48	– 0.001	– 3: 0.17 (0.04–0.70) 4: 0.23 (0.05–0.94)	– 6.38	– 0.04

OR, odds ratio; CI, 95% Confidence interval; MIZ, maximal injury zone.

Categories of echogenicity‐MIZ: Reference = Anechoic; 1 = Hypoechoic.

Categories of CSA‐MIZ (% of total tendon cross‐sectional area affected): Reference = <25%; 1 = ≥25–50%; 2 = ≥50–75%, 3 = ≥75%.

Categories of LFP‐MIZ (% disruption of longitudinal fibre pattern): 0 = 0%; 1 = <25%; 2 = ≥25–50%; 3 = ≥50–75%; 4 = ≥75%.

a Since only n = 1 LFP‐MIZ of <25% the reference category was considered ≥25–50%.

b Statistical model tested individual effect, after correction for age and number of starts prior to the injury.

**Table 3:** Wald Prediction Values for successfully return to racing (completing ≥5 races) from 469 horses with SDFT injury in Hong Kong (2003–2014)

Percentage of tendon affected	SDFT tendonitis without core lesion (n = 112)		SDFT tendonitis with core lesion (n = 357)	
	Longitudinal Fibre Pattern‐MIZ^a^		Cross Sectional Area‐MIZ^a^	
	Prediction	95% CI	Prediction	95% CI
<25%	0.99	–	0.35	0.29–0.42
≥25–50%	0.70	0.50–0.82	0.29	0.19–0.35
≥50–75%	0.49	0.30–0.63	0.16	0.06–0.28
≥75%	0.14	0.14–0.36	0.11	0.10–0.40

CI, 95% Confidence interval.

a MIZ, Maximal injury zone.

## Discussion

This is the first study to describe how specific features from the first ultrasonographic examination of a forelimb SDFT injury can be used to predict likely long‐term outcome for that racehorse (successfully completing ≥5 races after injury). In particular, cases in which a high proportion of the tendon CSA was affected (≥50%) or where there was significant disruption of the LFP (≥75%) had a reduced likelihood of making a successful return to racing. In accordance with previous research, completion of five or more races is considered an appropriate indicator for successful treatment and return to productive performance 13.

Our finding that the right forelimb was injured more often than the left is in agreement with previous literature 2. This may reflect that training and racing at the HKJC is mainly in a clockwise direction. In contrast, the left forelimb was found to be more commonly affected in horses worked in an anticlockwise direction 14. These findings suggest that the inside limb, which tends to be the lead limb at the gallop around a bend 15, is predisposed to injury. This cannot simply be explained by the magnitude of loads as the ground reaction force is highest in the nonlead limb at the canter and is predicted to be similar at higher speeds 16. Further biomechanical studies are required to elucidate potential mechanisms behind this risk factor.

In this study, the prevalence of bilateral SDFT pathology was less than 10%, which is in contrast to National Hunt (NH) racehorses in the UK where both forelimbs are affected in 35% of cases 17. Shorter races, different loads in the absence of jumps and younger horses may account for this variation. Previous studies have found that the risk of injury increases with age 18, which was explained by age‐related changes in the tendon matrix. Conversely, this study found that the number of SDFT injuries increased gradually up to the age of 4 years old and then decreased. However, data were not analysed in relation to the total number of horses racing/training at different ages and therefore the prevalence of SDFT tendonitis in this population was not assessed. In addition, some horses without a complete initial ultrasonographic examination were excluded. Male sex has also been previously identified as an associated risk factor for SDFT injury 19. Unfortunately, this could not be corroborated in this study as almost all included horses were male. Currently, evidence is contradictory about the effect of racing (number of starts) prior to the injury. Tendon pathology may accumulate during a horse′s racing career, which could increase the risk of injury 20, although this is confounded with increasing age. On the other hand, one study has shown that the number of races completed before injury is associated with a decrease in the risk of tendon injury, suggesting a protective effect. Musculoskeletal tissue remodelling following racing/training could lead to tendons that are more resistant to damage 21.

In the current study, the MIZ was the mid‐metacarpus region (i.e. zones 2B and 3A), which is in accordance with previous reports 22. Despite cross‐sectional area of the SDFT being lowest at this location, the tendon is not significantly weaker 23. However, blood supply to the tendon matrix is minimal in the mid metacarpus region, which may predispose the tendon to hypoxia 23 and/or hyperthermia during exercise 24. This may result in localised necrosis and degeneration of matrix, which may predispose to injury.

When present, core lesions were more commonly located centrally (53%) than towards the periphery (39%) of the tendon, which is in agreement with previous studies 8. Core lesions may be related to pre‐existing microdamage 8 or degeneration 25, which is more likely to occur in the central region of the tendon during high‐speed locomotion. Nevertheless, there was no statistical association between the location of the core lesion at the MIZ and successful return to racing. In cases with a core lesion, the proportion of the cross‐sectional area of the tendon affected at the MIZ was related to the likelihood of a successful return to racing as in previous studies 14, 26. The proportion of the cross‐sectional area of the tendon affected has been suggested as the most objective measure of tendon pathology as it can be assessed accurately using ultrasonography and it is a relative measure and, as such, less affected by variation in technique 27. Cases that had both peripheral and central core lesions at the MIZ, were significantly less likely to race again, which may simply reflect the fact that the overall proportion of the tendon cross‐sectional area affected was greater.

In cases with tendonitis without an obvious core lesion, the percentage of cross‐sectional area affected at the MIZ was not a useful predictor of return to racing. However, the injuries in the majority of the horses in this group did not affect a high percentage of the tendon′s cross‐sectional area, giving low statistical power to this US parameter. The percentage of longitudinal fibre pattern affected at the MIZ was found to be the strongest factor influencing a successful return to racing in these cases.

Reduced echogenicity (hypoechoic or anechoic) represented an important, and significant factor influencing the likelihood of a successful return to racing in both groups. However, this US parameter should be considered a confounding factor for both Groups 1 and 2, since all acute injuries are, by definition, either hypoechoic or anechoic and all cases in this study were acute (assessed by US at the time of injury). The hypoechoic or anechoic appearance of acute SDFT lesions represents haemorrhage, oedema and early granulation of tissue within the lesion 28. There were no hyperechoic lesions in this study.

There is some discussion that cases of SDFT tendonitis with a discrete core lesion may represent more severe injuries than those without a core lesion. However, this study found that in cases that successfully return to racing, the average time to first start and the number of races following injury was similar between the two types of injury. Both types of injury should therefore be considered equally important.

A potential limitation of this study is operator and equipment related variability 27. Ultrasonographic exams were obtained over a 10‐year period by several different clinicians using different ultrasound machines. However, all ultrasonographic images were analysed by a single person (R.A.) in this study using an ultrasonographic scoring system that has been previously validated 12, allowing for a more objective, reliable and repeatable assessment of all images. Although no attempt was made to assess or compare the quality of the images, which is also a potential limitation of this retrospective study, the test conditions throughout the study were kept consistent. The ultrasonographic scoring system 12 used in this study had been previously validated by experienced clinicians using only clinical cases from this population and included cases from each year of study. In this study, the date of the first scan was used as the date of the injury. It is acknowledged, however, that a majority of horses may have sustained the injury a few days before the initial exam, as a fixed protocol for reporting of SDFT injury was not in place. Nevertheless, this minor difference in time of injury should not materially affect our primary outcome. Indeed, performing US examinations too soon after injury can result in an underestimation of injury severity, as SDFT injuries tend to worsen within the first 7 days 7.

The range of treatments used for SDFT injury in this study were varied and beyond the control of the investigators, since we were conducting a retrospective analysis. However, as a potential confounder for our primary outcome we considered the effect of differing treatments. Ideally, if a severe injury is present then the average time to retirement should be less than 7 days. Reaching this decision between the owner, trainer and veterinarian could sometimes take significantly longer. Therefore, for this part of the study, the authors have excluded all the horses that were retired within the first 28 days post‐injury. We suggest that the current ultrasonographic study can help inform that evidence‐base such that other reasons like a horse's ability/performance, value, concomitant maladies or age are secondary to simple facts about the nature and extent of the lesion itself. As a result, reaching any decision that could once take many days and only increase the retirement time of some of those horses and reduce their welfare could be made almost immediately at the time of initial examination. For this study, horses retired within the first 28 days following SDFT injury were considered retired straight away without any attempt to return to training/racing. This explains that the average retirement time for horses that were retired straight away in this study is 12 and 6 days for Groups 1 and 2 respectively. There were not enough cases included in the study to perform valid statistical analysis to evaluate intralesional therapies or surgical treatment. The confidence intervals for those treatments analysed were wide, indicating low sample size with high variability. Based on these results, it is unlikely that any of the assessed treatments influenced the results of this study. Indeed, when included as potential confounders in a logistic regression model, our main outcomes remained significant. Reaching agreement among veterinarian, owners and trainers is always difficult as each have differing priorities (i.e. clinical, financial and practical issues are involved). Variation in healing and subsequent success in racing could potentially be a confounding factor in this study due to variation in treatment, amount of rest and the type of rehabilitation.

The study population reported here is unique and highly controlled, in that it includes almost entirely male racehorses that train and compete in a clockwise direction on the same dirt or turf surface, respectively. Therefore, these predictive factors should be used with caution in other sporting horse populations (i.e. eventers, showjumpers or Thoroughbred racehorses racing in other countries) as different factors might be involved. Future studies are warranted to characterise SDFT injury in other sport disciplines.

This study presents robust evidence that ultrasonography has value in characterising the extent and nature of SDFT injuries. It also provides data on predictive factors based on simple ultrasonographic measures which can be easily applied in equine practice. The key factors at the site of maximal injury were the extent of cross‐sectional area affected in cases with a core lesion, or the severity of longitudinal fibre pattern disruption in cases without a core lesion. All assessments were made at the initial ultrasound examination of the horse. Our data, therefore, will enable equine clinicians to make early evidence‐based decisions on SDFT injury. This will significantly affect racehorse welfare in the short‐ and long‐term, as it provides a scientific basis for owners and trainers to make timely decisions, and consider early retirement or alternative career options for specific horses.

## Authors’ declaration of interests

The authors have no competing interests.

## Ethical animal research

This study was reviewed and approved by the ethical committee of the School of Veterinary Medicine and Science, University of Nottingham. Data was retrieved from records and horse identifiers were removed by HKJC staff. Owner consent to the use of retrospectively collected data was indicated by the acceptance of owners of the Rules of Racing of the Hong Kong Jockey Club (under rule 46.1 and 46.2) on the granting of their racing permit.

## Sources of funding

This study has received funding support or support in kind from Oakham Veterinary Hospital and the Hong Kong Jockey Club.

## Authorship

R. Azola was the main researcher, with the main responsibility for the study execution, data analysis and interpretation, and preparation of the manuscript. C. Easter conducted an initial study of 245 horses, developing the first study protocols including initial scoring system and data retrieval systems which informed the final study design with a larger study population over a longer time duration. D. Gardner, C. Riggs and S. Freeman were all responsible for the original study design and contributed to study execution, data analysis and interpretation, and manuscript preparation throughout. In addition, C. Riggs coordinated data retrieval and D. Gardner was responsible for statistical methodology. All authors contributed to and approved the final manuscript.

## Supporting information


**Supplementary Item 1:** Age at injury in 469 flat racehorses with SDFT injury in Hong Kong (2003–2014). Compulsory retirement age at the HKJC (10‐year‐old).

**Supplementary Item 2:** Limb affected in 469 flat racehorses with SDFT injury in Hong Kong (2003–2014).

**Supplementary Item 3:** Results of treatment options in 469 flat racehorses with SDFT injury in Hong Kong (2003–2014) excluding horses that were retired within the first 28 days post‐injury.

## Manufacturers' addresses

1 AGFA Healthcare, Brentford, Middlesex, UK.

2 VSN International, Hemel Hempstead, Hertfordshire, UK.
