# Supplementary material for: Ultrasonographic‐based predictive factors influencing successful return to racing after superficial digital flexor tendon injuries in flat racehorses: A retrospective cohort study in 469 Thoroughbred racehorses in Hong Kong
Source: Equine Vet J. 2018 Feb 23;50(5):602–8. doi: 10.1111/evj.12810 (PMC6099230; doi:10.1111/evj.12810)

Equine Veterinary Journal

Supplementary Item 1: Age at injury in 469 flat racehorses with SDFT injury in Hong Kong (2003-2014). Compulsory retirement age at the HKJC (10-year-old).

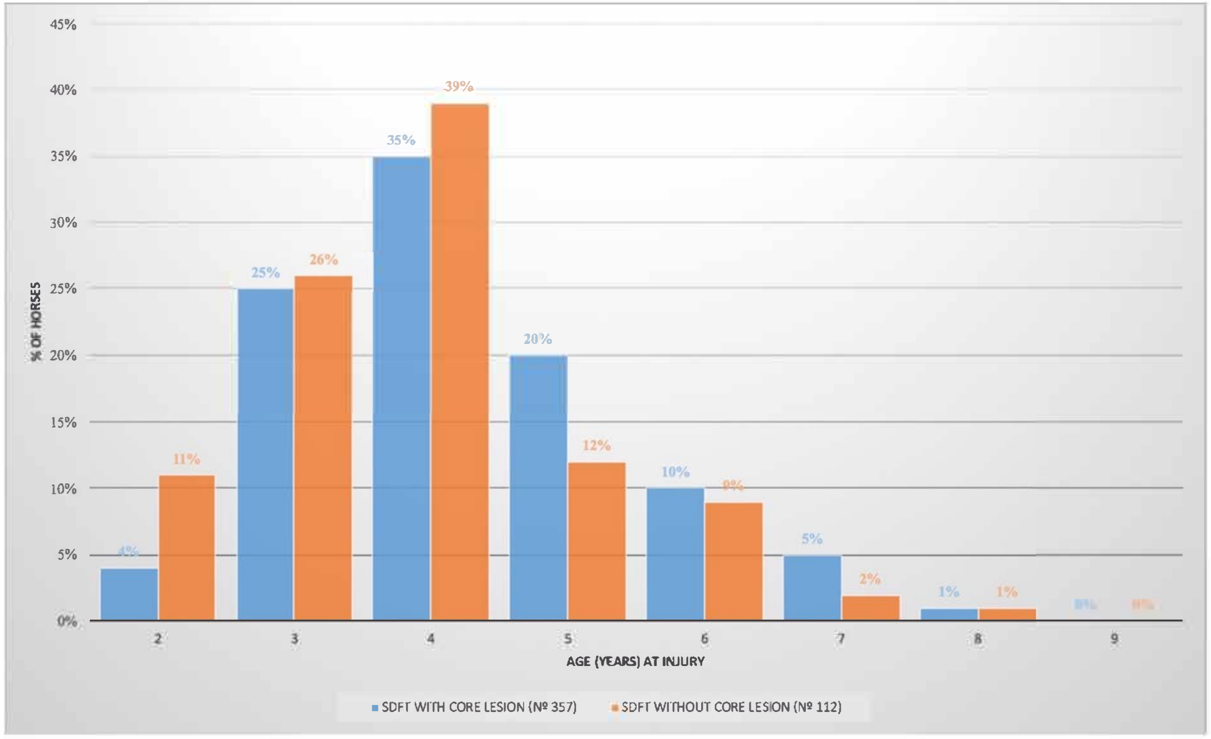

Supplement: Supplementary file 1 — Supplementary Item 1: Age at injury in 469 flat racehorses with SDFT injury in Hong Kong (2003–2014). Compulsory retirement age at the HKJC (10‐year‐old). [file EVJ-50-602-s001.pdf]
