# Supplementary figures and images for: Ultrasonographic‐based predictive factors influencing successful return to racing after superficial digital flexor tendon injuries in flat racehorses: A retrospective cohort study in 469 Thoroughbred racehorses in Hong Kong
Source: Equine Vet J. 2018 Feb 23;50(5):602–8. doi: 10.1111/evj.12810 (PMC6099230; doi:10.1111/evj.12810)

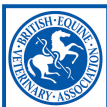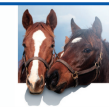

**Supplementary Item 2:** Limb affected in 469 flat racehorses with SDFT injury in Hong Kong (2003-2014).

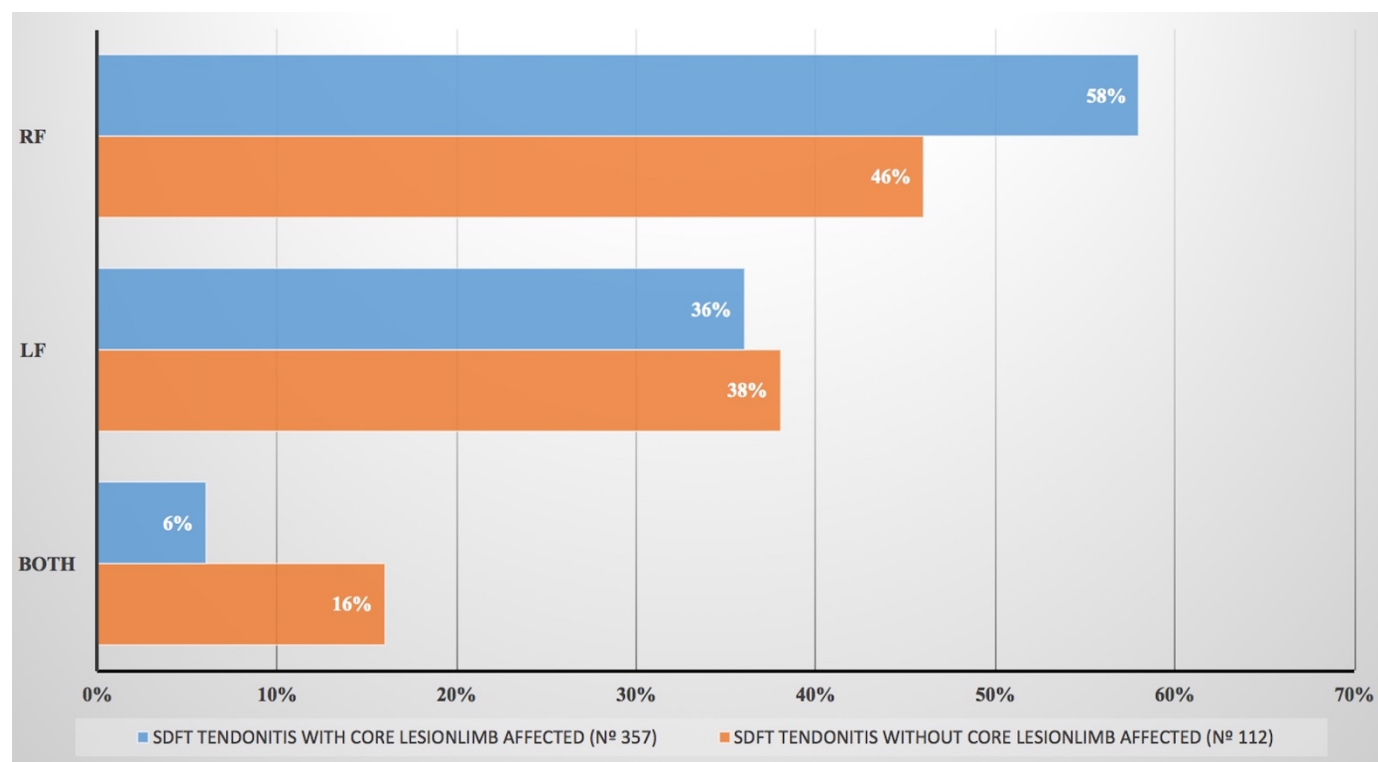

Supplement: Supplementary file 2 — Supplementary Item 2: Limb affected in 469 flat racehorses with SDFT injury in Hong Kong (2003–2014). [file EVJ-50-602-s002.pdf]
