# Supplementary material for: Ultrasonographic‐based predictive factors influencing successful return to racing after superficial digital flexor tendon injuries in flat racehorses: A retrospective cohort study in 469 Thoroughbred racehorses in Hong Kong
Source: Equine Vet J. 2018 Feb 23;50(5):602–8. doi: 10.1111/evj.12810 (PMC6099230; doi:10.1111/evj.12810)
Supplement: Supplementary file 3 — Supplementary Item 3: Results of treatment options in 469 flat racehorses with SDFT injury in Hong Kong (2003–2014) excluding horses that were retired within the first 28 days post‐injury. [file EVJ-50-602-s003.pdf]

Supplementary Item 3: Results of treatment options in 469 flat racehorses with SDFT injury in Hong Kong (2003-2014) excluding horses that were retired within the first 28 days post-injury.

A note of caution in regard to these data: the study was originally powered and designed to test the primary outcome (successfully raced again after SDFT injury) rather than the efficacy of a particular treatment regimen. Hence, interpretation of secondary outcomes such as treatments for SDFT injury are not within the scope of this study. Therefore, these results should be interpreted with care. Further research is required to clarify the significance of these observations. Hence, for this analysis we assumed a more conservative significance level, adjusted for the number of comparisons, of P = <0.01

Group 1 (SDFT tendonitis with a core lesion): 21% of horses (n = 78/357) were retired within 28 days following injury and 79% received medical/surgical treatment. The average retirement time for horses that did not receive treatment following injury was 12 [4-18] days (median [IQR]). Ice therapy appeared to have a negative effect (95% CI 0.14-0.45; p<0.01) in contrast to shockwave therapy which appeared to have a positive effect (95% CI 1.36-4.13; p<0.01) on horses having a successful return to racing. All other treatments were deemed non-significant (i.e. p>0.01).

Group 2 (SDFT tendonitis without a core lesion): 19% of horses (n = 22/112) were retired within 28 days following injury and 81% of the horses received medical/surgical treatment. The average retirement time for horses that did not receive treatment following injury was 6 [2-15] days (median [IQR]). Blistering appeared to have a negative effect (95% CI 0.03-0.74, p = 0.01) on horses making a successful return to racing, with all other treatments having no significant (p>0.01) effect.

Effect of treatment on racehorses with SDFT injury (with [Group 1] or without [Group 2] core lesion)					
Variable		Univariate analysis		Multivariate analysis	
		OR (95% CI)	P-value	OR (95% CI)[1]	P-value
1.Treatment of Injury:	None	Ref: no treat	-	-	-
	Exercise	4.61 (0.56-37.7)	0.09	3.65 (0.41-32.4)	0.24
	NSAID	1.39 (0.84-2.30)	0.18	1.08 (0.60–1.93)	0.79
	Steroids	2.96 (0.17-7.47)	0.01	2.05 (0.72-5.84)	0.17
	Blistering	1.81 (0.95-3.45)	0.07	1.33 (0.64-2.76)	0.43
	Shockwave	3.20 (1.90-5.40)	**<.001**	2.35 (1.31-4.22)	**0.004**
	Ice therapy	0.25 (0.15-0.43)	**<.001**	0.44 (0.17-1.12)	**<.001**
2. Treatment of Injury:	None	Ref: no treat	-	-	-
	Exercise	3.19 (0.61-16.7)	0.13	2.19 (0.53-9.03)	0.27
	NSAID	1.79 (1.26-2.54)	< .001	1.36 (0.59–3.10)	0.45
	Steroids	0.78 (0.13-4.66)	0.79	0.67 (0.18-2.42)	0.65
	Blistering	1.14 (0.31-4.18)	0.86	0.70 (0.21-2.35)	0.64
	Shockwave	2.69 (1.00-7.18)	0.04	2.59 (1.05-6.42)	0.03
	Ice therapy	0.71 (0.25-2.02)	0.52	0.44 (0.17-1.12)	0.08
